# Protection from β-cell apoptosis by inhibition of TGF-β/Smad3 signaling

**DOI:** 10.1038/s41419-020-2365-8

**Published:** 2020-03-13

**Authors:** Ji-Hyeon Lee, Jose Manuel Mellado-Gil, Young Jae Bahn, Sushrut M. Pathy, Ying E. Zhang, Sushil G. Rane

**Affiliations:** 1 Cell Growth and Metabolism Section, Diabetes, Endocrinology, and Obesity Branch, National Institute of Diabetes and Digestive and Kidney Diseases, National Institutes of Health, Bethesda, MD USA; 2 Laboratory of Cellular and Molecular Biology, Center for Cancer Research, National Cancer Institute, National Institutes of Health, Bethesda, MD USA; 3 Present Address: Biomedical Research and Innovation Institute of Cádiz (INiBiCA) Research Unit, Jerez University Hospital, Cádiz, Spain

**Keywords:** Apoptosis, Type 2 diabetes

## Abstract

Prevailing insulin resistance and the resultant hyperglycemia elicits a compensatory response from pancreatic islet beta cells (β-cells) that involves increases in β-cell function and β-cell mass. However, the sustained metabolic stress eventually leads to β-cell failure characterized by severe β-cell dysfunction and progressive loss of β-cell mass. Whereas, β-cell dysfunction is relatively well understood at the mechanistic level, the avenues leading to loss of β-cell mass are less clear with reduced proliferation, dedifferentiation, and apoptosis all potential mechanisms. Butler and colleagues documented increased β-cell apoptosis in pancreas from lean and obese human Type 2 diabetes (T2D) subjects, with no changes in rates of β-cell replication or neogenesis, strongly suggesting a role for apoptosis in β-cell failure. Here, we describe a permissive role for TGF-β/Smad3 in β-cell apoptosis. Human islets undergoing β-cell apoptosis release increased levels of TGF-β1 ligand and phosphorylation levels of TGF-β’s chief transcription factor, Smad3, are increased in human T2D islets suggestive of an autocrine role for TGF-β/Smad3 signaling in β-cell apoptosis. Smad3 phosphorylation is similarly increased in diabetic mouse islets undergoing β-cell apoptosis. In mice, β-cell-specific activation of Smad3 promotes apoptosis and loss of β-cell mass in association with β-cell dysfunction, glucose intolerance, and diabetes. In contrast, inactive Smad3 protects from apoptosis and preserves β-cell mass while improving β-cell function and glucose tolerance. At the molecular level, Smad3 associates with Foxo1 to propagate TGF-β-dependent β-cell apoptosis. Indeed, genetic or pharmacologic inhibition of TGF-β/Smad3 signals or knocking down Foxo1 protects from β-cell apoptosis. These findings reveal the importance of TGF-β/Smad3 in promoting β-cell apoptosis and demonstrate the therapeutic potential of TGF-β/Smad3 antagonism to restore β-cell mass lost in diabetes.

## Introduction

Pancreatic islet beta cells (β-cells) secrete insulin in response to rising glucose levels and deficit of β-cell mass underlies both forms of diabetes^1,2^. Autoimmune destruction of β-cells characterizes type 1 diabetes. Severe β-cell dysfunction and progressive loss of β-cell mass are the principal features of β-cell failure seen in T2D^3^. New observations continue to shed light on the regulation of β-cell mass and the potential approaches to regenerate β-cell mass in diabetes^4,5^. Pancreatic development involves waves of endocrine and exocrine pancreas growth and morphogenesis that includes dynamic changes to β-cell mass^6–8^. In addition, changes to β-cell mass are seen in normal physiological adaptations occurring during aging and pregnancy, during pathological states such as obesity and in response to toxins or regeneration stimuli^9^. β-cell proliferation has been a focus of most rodent studies investigating β-cell mass, with replication of pre-existing β-cells and neogenesis from a progenitor pool within the pancreatic ductal epithelium regarded as the two main sources of increased β-cell mass^10–12^. The role of β-cell dedifferentiation, as it relates to changes in β-cell mass, is relatively unclear and is an active area of investigation^13,14^.

β-cell apoptosis has been observed in rodent and human pancreas^1,15–19^. However, in comparison to our knowledge of β-cell proliferation^10–12^, the process of β-cell apoptosis is incompletely understood^20^. A landmark study of human cadaveric pancreas underscored the role of apoptosis in the observed deterioration of β-cell mass in T2D, regardless whether the T2D subjects were lean or obese^15^. In that same study, the levels of β-cell replication and neogenesis within the cadaveric human T2D pancreas were low and indistinguishable when compared with nondiabetic (ND) human pancreas specimens. Thus, the observations of Butler and colleagues were consistent with apoptosis being one of the primary mechanisms responsible for the reduced β-cell mass in human T2D pancreas. From a mechanistic viewpoint, high glucose or fatty acids stimulate β-cell apoptosis that appears to involve endoplasmic reticulum stress, amyloid deposition, ceramide and reactive oxygen pathways, and mitochondrial dysfunction^16,21–28^. However, the role of extracellular signaling pathways in β-cell apoptosis is largely obscure^20^.

We and others have described the role of TGF-β superfamily signaling in endocrine pancreas development and β-cell proliferation and differentiation^29–35^. Considering its established role in cellular apoptosis, especially within the context of complex human diseases such as cancer^36^, we hypothesized that TGF-β signaling might play a similar role in β-cell apoptosis during T2D pathogenesis. Our observations are supportive of an autocrine role for TGF-β signaling in β-cell apoptosis. We show that constitutively-active TGF-β/Smad3 signals promote whereas inhibition of TGF-β/Smad3 signals protect from β-cell apoptosis.

## Results

### Increased TGF-β/Smad3 signaling correlates with high-fat diet (HFD)-induced β-cell apoptosis

**Fig. 1:**
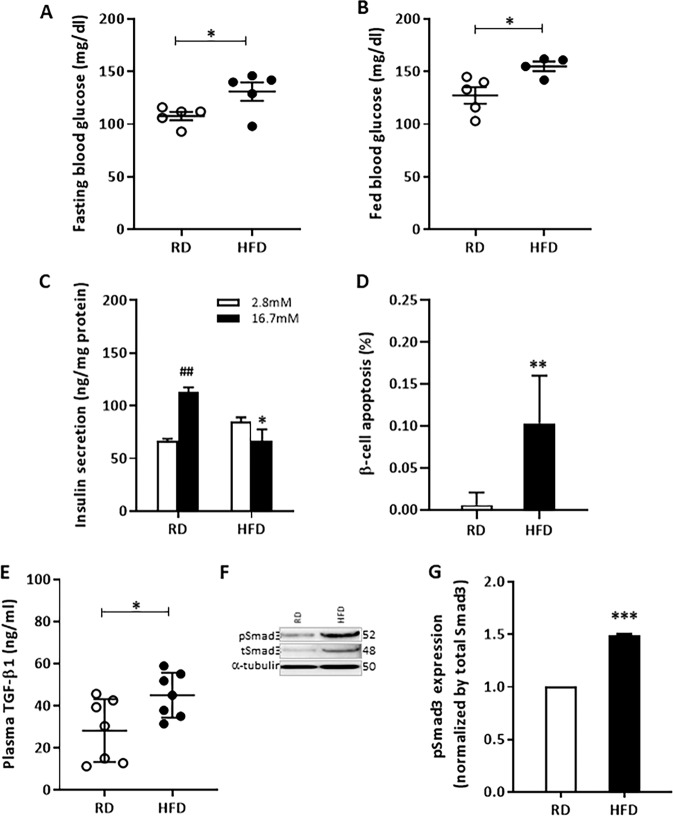
Activation of TGF-β1/Smad3 signaling is associated with HFD-induced β-cell dysfunction and apoptosis. Male C57BL/6J mice (*n* = 5 per group) fed high-fat diet (HFD) or regular diet (RD) for 16 weeks, starting from 6 weeks old age, were used in all experiments below. Daytime (6 h, 8 am–2 pm) fasting glucose (**a**) and morning-fed glucose (**b**) are shown. **c** Islets from RD or HFD-fed male mice were isolated and ten handpicked similar sized islets were incubated with low (2.8 mM) or high (16.7 mM) glucose for 1 h at 37 °C and the media were collected for the insulin secretion assay. Insulin secretion level in the media was normalized to total protein from each group of ten islets (*n* = 3 replicates per mouse, three mice per group). **d** Pancreatic sections (*n* = 3 mice per group) were fixed in formalin and stained with TUNEL and insulin for β-cell apoptosis assessment. TUNEL+ insulin+ β-cells were counted and divided by total islet β-cell (insulin+) number and presented as % of β-cell apoptosis. Minimum 3000 islet β-cells were counted per mouse. **e** Fed state plasma TGF-β1 levels were measured from the RD and HFD-fed mice (*n* = 7 per group). **f** Activation of TGF-β/Smad3 signaling was evaluated by examining levels of pSmad3 expression in islets pooled from three mice in each group (*n* = 2 replicates). **g** pSmad3 expression was normalized to total Smad3 expression and presented as fold change. Data are mean ± SD. *p < 0.05, **p < 0.01, ***p < 0.001 vs. RD; ^##^*p* < 0.01 vs. 2.8 mM glucose by two-side, unpaired Student *t* test.

To better understand the underlying mechanisms responsible for β-cell apoptosis, we utilized a mouse model system where metabolic stress was induced via extended feeding of HFD. Normal 6-week-old male C57BL6 mice were fed either a regular diet (RD) or 60% HFD for 16 weeks and their glycemic levels were monitored. Fasting and fed-state hyperglycemia observed in the HFD-fed mice (Fig. 1a, b) were associated with increased body weight and glucose intolerance (Supplementary Fig. 1a, b). Further, HFD feeding induced β-cell dysfunction as evidenced by impaired glucose-stimulated insulin secretion (GSIS) in isolated islets (Fig. 1c). Increased TUNEL^+^ insulin^+^ cells in pancreatic sections (Fig. 1d) and in dispersed islet β-cell cultures (Supplementary Fig. 1c) from HFD-fed mice were indicative of increased susceptibility to HFD-induced β-cell apoptosis. The gradual process of apoptosis makes it difficult to detect β-cell death in pancreas tissue sections. Furthermore, the TUNEL staining protocol detects cells in later stages of cell death with the dead cells being scavenged immediately. To complement the data using pancreas sections (Fig. 1d), we thus utilized a dispersed islet β-cell model to evaluate the susceptibility of β-cell death (Supplementary Fig. 1c). This model allows better assessment of cell death susceptibility under various experimental conditions and in response to defined stimuli. Such information is difficult to acquire using solely in vivo models, due to lack of knowledge of the kinetics of the cell death process and the need for greater numbers of mice to achieve statistically significant data. Plasma levels of TGF-β1 were increased in HFD mice (Fig. 1e) along with increased total and phospho-Smad3 expression in the HFD islets compared with RD islets (Fig. 1f, g). We next set up experiments to inquire whether the deregulated TGF-β signals contribute to the β-cell apoptosis associated with the HFD feeding regimen.

### Mouse models with β-cell-specific expression of constitutively-active or -inactive Smad3

**Fig. 2:**
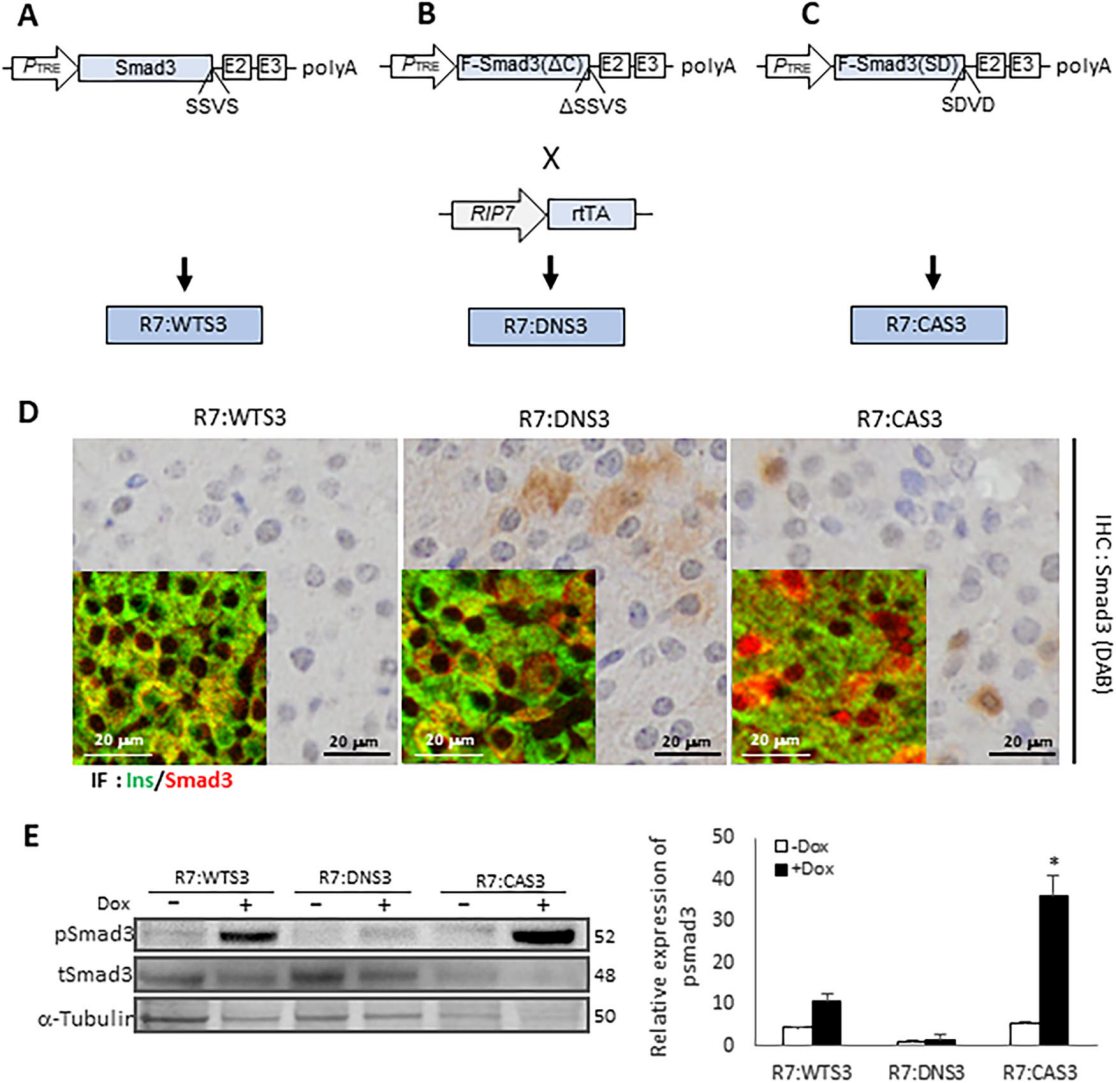
Expression of Smad3 transgenes in β-cells. TRE-Smad3 mice expressing (**a**) wild-type Smad3 (Smad3), **b** dominant-negative Smad3 lacking the last four amino acid residues (Smad3 ΔC), and **c** constitutively-active Smad3 with the last two serines substituted by aspartic residues to mimic phosphorylation (Smad3 SD) via the tetracycline-responsive element (TRE). Pancreas β-cell-specific expression of transgenes was achieved by breeding the TRE-Smad3 mice with RIP7-rtTA mice, which express reverse tetracycline-controlled transactivator (rtTA) specifically in β-cells under the control of the rat insulin II promoter (RIP7). The resulting double transgenic mice were designated as R7:WTS3, R7:DNS3, and R7:CAS3, respectively. **d** Smad3 expression was evaluated in pancreatic sections from 4-month-old transgenic mice-administered doxycycline-containing diet (200 mg/kg) for 2 months. Formalin-fixed pancreatic sections were stained with Smad3 antibody (shown in brown) in immunohistochemistry assays and insulin (green) and Smad3 (red) double immunofluorescence assays (inset). **e** Islets were isolated from 4-month-old Smad3 transgenic mice without or with doxycycline (Dox) diet-administration for 2 months and assessed for expression of pSmad3 levels by western blot analyses. pSmad3 expression was normalized to total Smad3 expression and presented as relative pSmad3 expression in the graph. *p < 0.05 vs. Dox by Student’s *t* test.

To investigate the role of TGF-β/Smad3 in β-cell apoptosis, we developed mice with β-cell-specific expression of transgenes expressing wild-type (WT), constitutively-active (CA), or dominant-negative (DN) Smad3 (Fig. 2a–c). WT Smad3 is activated via phosphorylation by TGF-β receptor 1 (TβR1) kinase on serine residues located on amino acids Serine-Serine-Valine-Serine (SSVS) at the C-terminus. Deletion of amino acids SSVS precludes phosphorylation of the serine residues by TβR1, thereby generating a DN Smad3. In contrast, amino acid substitution of the SSVS to Serine-Aspartic Acid-Valine-Aspartic Acid (SDVD) mimics phosphorylation, rendering Smad3 constitutively active (CA Smad3) and thus independent of TβR1 kinase activity. In addition, we engineered a Tetracycline responsive element (TRE) into the constructs which allows time-conditional transgene expression when a tetracycline analog, doxycycline (Dox), is administered via diet. Mice harboring these transgenes are referred to as TRE-WT Smad3, TRE-DN Smad3, and TRE-CA Smad3 mice. For β-cell-specific transgene expression, these mice were bred to mice expressing the reverse-tetracycline transactivator (rtTA) under the control of a rat insulin promoter (RIP7 or R7) to generate rtTA-RIP7:TRE-WT Smad3 (R7:WTS3), rtTA-RIP7:TRE-DN Smad3 (R7:DNS3), and rtTA-RIP7:TRE-CA Smad3 (R7:CAS3) mice (Fig. 2a–c). In these mice, the respective Smad3 transgenes will be expressed in β-cells when the rat insulin promoter activation occurs in the presence of Dox. Transgene activation will occur early in development when Dox is delivered in utero to the pups via pregnant mothers ingesting a diet containing Dox. Alternatively, activation of the transgenes in adult mice would occur at defined time windows when mice of specific ages are fed a Dox-containing diet.

Immunohistochemical and immunofluorescence analyses revealed increased nuclear Smad3 expression in β-cells from pancreas harvested from Dox-fed R7:CAS3 mice indicating the presence of activated Smad3 (Fig. 2d). In contrast, predominant cytoplasmic Smad3 expression was seen in β-cells from R7:DNS3 pancreatic section, consistent with primarily inactive Smad3. In agreement, western blot analyses confirmed that levels of phosphorylated Smad3 were increased in islets isolated from Dox-fed R7:CAS3 mice and those levels were suppressed in R7:DNS3 islets, compared with levels of phosphorylated Smad3 observed in islets from Dox-fed R7:WTS3 mice (Fig. 2e). These mice thus enable investigation into the effects of constitutively-activating or -inhibiting TGF-β/Smad3 activity in islet β-cells at defined times during embryonic development and postnatal adult stages.

### β-cell dysfunction, glucose intolerance, and diabetes in R7:CA Smad3 mice; improved β-cell function and enhanced glucose tolerance in R7:DN Smad3 mice

**Fig. 3:**
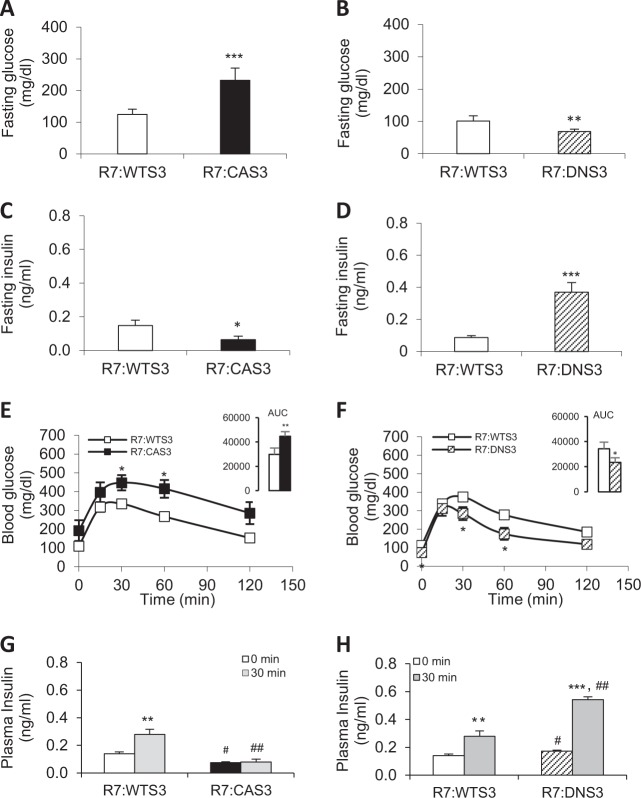
CA Smad3 causes β-cell dysfunction, glucose intolerance, and diabetes, whereas, DN Smad3 improves β-cell function and glucose tolerance. Transgene activation during early development was initiated via doxycycline (200 mg/kg) delivered in utero to progeny by pregnant mothers ingesting diet containing doxycycline and the progeny was continuously administered the Dox diet until further analysis. Overnight 16 h fasting glycemia (**a**, **b**) and plasma insulin level (**c**, **d**) from 2 months old transgenic male mice were measured. **e**, **f** IPGTT was performed in 2-month-old male mice and AUC was determined (see inset). **g**, **h** Insulin levels at 0 and 30 min after glucose (2 g/kg) injection were measured. All results are expressed in means ± SD of 5–7 mice per group. *p < 0.05, **p < 0.01, ***p < 0.001 vs. R7:WTS3 (**a–f**) and **p < 0.01, ***p < 0.001 vs. 0 min; ^#^*p* < 0.05, ^##^*p* < 0.01 vs. R7:WTS3 (**g–h**) by unpaired Student^’^s *t* test.

Fasting glucose levels and glucose tolerance in Dox-administered R7:WTS3 mice were similar to those observed in Dox-administered TRE-Smad3 alone or rtTA-RIP7 alone control mice (Supplementary Fig. 2a, b). Levels of glycemia were next tested in transgenic mice with or without Dox (Supplementary Fig. 2c). Fasting glycemia in R7:CAS3 mice without Dox trended to be higher than that seen in R7:WTS3 mice without Dox administration. Levels of fasting glucose upon Dox administration were higher in R7:CAS3 mice and lower in R7:DNS3 mice, compared with levels observed in R7:WTS3 mice (Supplementary Fig. 2c). For subsequent analyses, mice born to mothers fed Dox-containing RD during pregnancy were maintained on a Dox-diet regimen post birth and characterized at two months of age. R7:CAS3 mice exhibited fasting hyperglycemia (232.0 ± 38.6 mg/dl), compared with that seen in the R7:WTS3 (125.0 ± 15.9 mg/dl) mice, whereas the R7:DNS3 (68.4 ± 7.8 mg/dl) mice displayed lower fasting glucose levels when compared with their R7:WTS3 cohort (101.7 ± 15.8 mg/dl) (Fig. 3a, b). These levels of fasting glycemia were similar to those seen in double transgenic mice (R7:DNS3 or R7:CAS3) when compared with either TRE-Smad3 or rtTA-RIP7 control mice-administered Dox (Supplementary Fig. 2a). Taken together, these results demonstrated that glycemic states were indistinguishable in the R7:WTS3 mice and either TRE-Smad3 or rtTA-RIP7 control mice-administered Dox. For subsequent analyses, we thus utilized the R7:WTS3 mice as controls to characterize the phenotype of R7:DNS3 and R7:CAS3 mice. Fasting insulin levels were suppressed in the R7:CAS3 mice (Fig. 3c), while they were elevated in the R7:DNS3 mice, although within normal ranges (Fig. 3d). Glycemia and insulin levels in female transgenic mice were comparable to those observed in genotype-matched male transgenic mice (data not shown). Further, we observed impaired glucose tolerance in the R7:CAS3 mice with higher glucose levels during the 2-h glucose tolerance test (GTT) along with a significantly higher area under the curve (AUC) during the entire GTT (Fig. 3e). In contrast, R7:DNS3 mice exhibited improved glucose tolerance with significantly lower glucose levels and a lower AUC during the 2-h GTT (Fig. 3f). We next monitored β-cell function in these mice by performing in vivo GSIS assays. As expected, the R7:WTS3 mice showed normal GSIS after a 30-min glucose administration (Fig. 3g). However, the R7:CAS3 mice failed to respond to glucose stimulation in the GSIS assay indicative of impaired β-cell function (Fig. 3g). In contrast, significantly improved GSIS was observed in the R7:DNS3 mice, consistent with improved β-cell function in these mice (Fig. 3h).

### CA Smad3 promotes whereas DN Smad3 protects from β-cell apoptosis

**Fig. 4:**
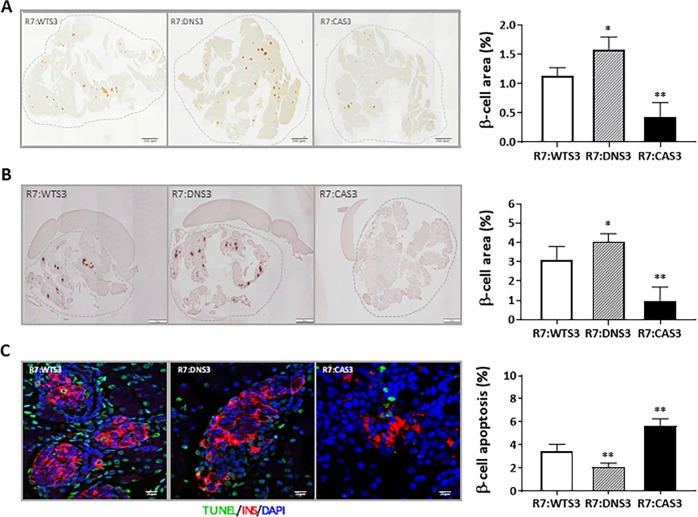
CA Smad3 promotes whereas DN Smad3 protects from β-cell apoptosis. Whole pancreatic sections from 4-month-old male mice (**a**) and at postnatal day 1 (**b**) from embryonic activation study were stained for insulin and β-cell area was detected using the DAB kit (shown in brown). % of β-cell area was calculated by normalization of total β-cell area to whole pancreatic section area (within dotted lines) in each image acquired and at least three inconsecutive sections per mouse and four mice from each group were used. **c** Pancreatic sections at postnatal day 1 from embryonic activation study were stained with insulin (red) and TUNEL (green) for quantitation of β-cell apoptosis. Total number of TUNEL+ β-cell was normalized to total β-cell number and data were presented as % of β-cell apoptosis. At least 3000 β-cells were counted per animal and at least 3 animals per group were used. *p < 0.05, **p < 0.01 vs. R7:WTS3.

β-cell area was significantly reduced in the R7:CAS3 pancreas at 4 months of age compared with R7:WTS3 pancreas, while β-cell area was increased in the age- and sex-matched R7:DNS3 pancreas (Fig. 4a). To investigate the primary mechanisms responsible for differences in β-cell mass, we examined pancreas from mice at postnatal day 1 (PD1). β-cell area was increased in the PD1 R7:DNS3 pancreas and decreased in the PD1 R7:CAS3 (Fig. 4b). TUNEL staining (Fig. 4c) and cleaved caspase-3 staining (Supplementary Fig. 3a) showed that levels of β-cell apoptosis were significantly increased in the PD1 R7:CAS3 pancreas, whereas those levels were significantly reduced in the PD1 R7:DNS3 pancreas. Levels of β-cell proliferation were reduced in PD1 R7:CAS3 pancreas and increased in the PD1 R7:DNS3 pancreas (Supplementary Fig. 3b). Together, these findings show that constitutive activation of TGF-β/Smad3 signaling during early pancreas development reduces β-cell mass in the adult pancreas. In contrast, inhibition of TGF-β/Smad3 signaling during early pancreas development enhances β-cell mass in the adult pancreas. Furthermore, these findings demonstrate that apoptosis is an important mechanism orchestrating the dynamic β-cell mass changes during pancreas development.

We next investigated the role of TGF-β/Smad3 signaling in adult β-cells. To this end, pregnant mothers were spared Dox in their diet and the pups were born without transgene activation in utero, thereby allowing pancreas development without perturbation of TGF-β/Smad3 signaling. To activate the transgenes in adult stages, mice were fed Dox-containing diet at different ages to activate or inhibit Smad3 at desired time points in adult β-cells. Levels of fasting glucose, GSIS, and glucose tolerance were measured to evaluate the effects of constitutively-activating or -suppressing Smad3 activity in adult β-cells. Even though a modest change in blood glucose levels and glucose tolerance was observed when 1-month-old mice were fed a Dox diet for a period of 4 months, no significant differences in glycemia, glucose tolerance, and GSIS were seen when mice between ages 2 months and 8 months were fed Dox diet (for a period of 4 months) to activate the Smad3 transgenes in β-cells (data not shown).

**Fig. 5:**
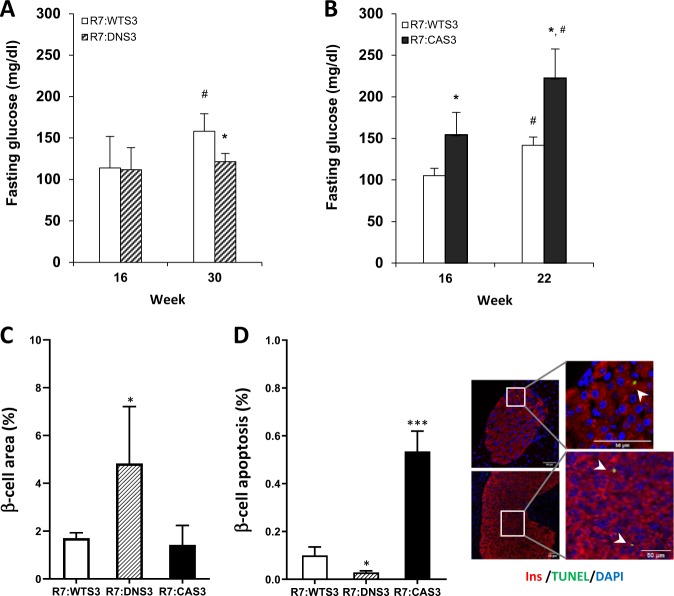
CA Smad3 induced hyperglycemia and increased β-cell apoptosis upon metabolic challenge in adult mice. Two-month-old male mice (*n* = 4–5 each per group) were fed 60% HFD supplemented with 200 mg/kg doxycycline (HFD + dox) for 16, 22, and 30 weeks and overnight 16 h fasting glycemia (**a**, **b**) was measured. After 16 weeks of HFD + dox feeding, pancreata were harvested, fixed and sections were examined for β-cell area by insulin staining (**c**) and for levels of β-cell apoptosis (**d**) using TUNEL (green) and insulin (red) co-staining protocols. % of β-cell area was calculated by determining total β-cell area with insulin staining in relation to whole pancreatic section area in each acquired section. **d** TUNEL positive β-cells among insulin-positive β-cells were counted and presented as % of β-cell apoptosis. At least three nonconsecutive sections per mouse and at least four mice from each group were used. Images show the representative pancreatic section from R7:CAS3 for TUNEL + β-cells (arrow). *p < 0.05, ***p < 0.001 vs. R7:WTS3; ^#^*p* < 0.05 vs. 16 weeks.

To investigate a potential role for Smad3 in adult β-cells facing a metabolic challenge, we repeated the above experiments using 2-month-old male transgenic mice that were fed 60% HFD supplemented with 200 mg/kg Dox (for up to 30 weeks). R7:DNS3 mice fed HFD for 16 weeks maintained normal fasting glucose levels, whereas fasting glucose was significantly elevated in the R7:CAS3 mice after 16 weeks of HFD feeding (Fig. 5a, b). Normal fasting glucose levels were detected in R7:DNS3 mice fed HFD up to 30 weeks, suggestive of extended protection from HFD-induced metabolic stress (Fig. 5a). In contrast, severe fasting hyperglycemia was observed in R7:CAS3 mice fed HFD for a comparatively shorter time of 22 weeks (Fig. 5b). Body weights were similar at 22 weeks of HFD + Dox feeding in all three lines (R7:WTS3 50.8 ± 1.5 g; R7:DNS3 48.3 ± 2.5 g; R7:CAS3 52.3 ± 4.3 g), discounting a role for body weight in the observed glucose levels. Impaired glucose tolerance was observed in R7:CAS3 mice whereas modest improvement in glucose tolerance was seen in R7:DNS3 mice, compared with that observed in R7:WTS3 mice (Supplementary Fig. 4a). We next investigated the effects of HFD feeding on β-cell mass and apoptosis by quantifying the insulin+ area for β-cell mass (Fig. 5c) and the TUNEL^+^:insulin^+^ apoptotic β-cells in pancreatic sections (Fig. 5d, representative TUNEL+:insulin+ staining sections are also shown). Increased β-cell mass with a significant reduction in levels of β-cell apoptosis were observed in R7:DNS3 mice fed HFD for 16 weeks (Fig. 5c, d). In contrast, higher levels of β-cell apoptosis were observed in islets from R7:CAS3 mice, although reduction in β-cell mass did not reach a level of significance (Fig. 5c, d). Taken together, these results are consistent with an important role for TGF-β/Smad3 signaling in promoting β-cell apoptosis induced by metabolic stress.

### Smad3 and FOXO1 interaction during β-cell apoptosis

**Fig. 6:**
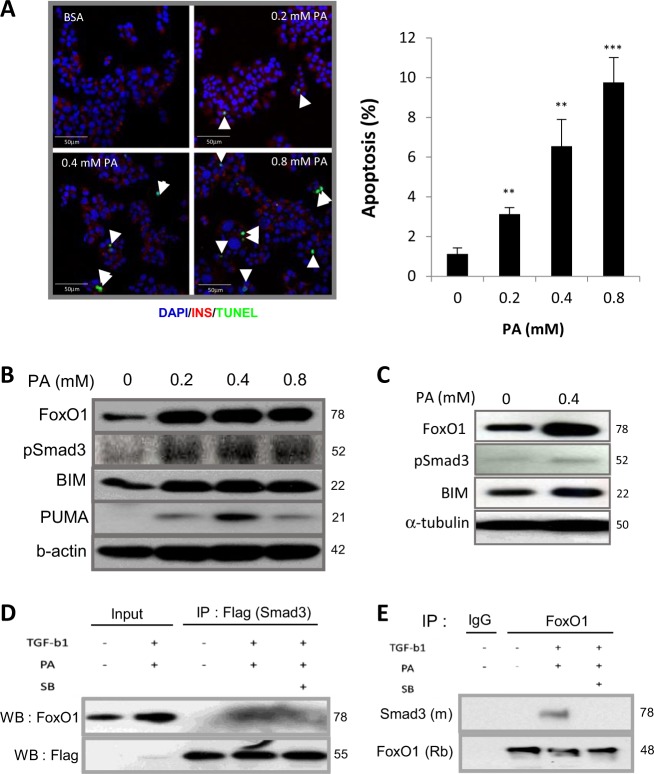
Elevated phospho-Smad3 and accumulation of FOXO1 during palmitate-induced β-cell apoptosis. **a** MIN6 cells were treated with various concentrations of palmitate (PA) for 24 h and β-cell apoptosis was evaluated by TUNEL staining (left panel, arrow head indicates TUNEL + MIN6 cells) and the number of TUNEL positive cells were divided by total number of cells in each treatment and presented as % of apoptosis (right panel, *n* = 3). **b**, **c** Expression of FOXO1, pSmad3, BIM, and PUMA were detected by western blot analyses in MIN6 cells (**b**) and normal mouse islets (**c**) after 24 h of palmitate treatment. **d**, **e** MIN6 cells transfected with Flag-tagged WT Smad3 expression plasmid (**d**) and untransfected MIN6 cells (**e**) were treated with 5 ng/ml of TGF-β1 and 0.5 mM PA with or without 10 µM of SB431542 (SB) for additional 24 h and analyzed for exogenous (**d**) and endogenous (**e**) binding between Smad3 and FOXO1 by immunoprecipitation experiments with either mouse anti-Flag (**d**) or rabbit anti-FOXO1 (**e**) antibodies followed by immunoblotting with rabbit anti-FOXO1 (Cell Signaling, Danvers, MA, USA) or mouse anti-Smad3 (Abcam) antibody, respectively. 1% Bovine serum albumin (BSA) was used as vehicle control for 0 mM PA group. **p < 0.01, ***p < 0.001 vs. 0 mM PA.

To better understand the role of TGF-β in β-cell apoptosis, we utilized a β-cell line and a primary islet-based culture system. Fatty acids such as palmitate promote β-cell apoptosis via activation of multiple mechanisms that include generation of ceramide and reactive oxygen species, activation of the mitochondrial apoptosis pathway, damage to components of the insulin secretory machinery, and depletion of endoplasmic reticulum Ca2+ stores^20^. Treatment of the MIN6 β-cell line with different concentrations of palmitate resulted in dose-dependent increase in β-cell apoptosis evidenced by the increased numbers of Insulin^+^:TUNEL^+^ cells (Fig. 6a). Culture of MIN6 cells and primary mouse islets with palmitate resulted in an increase in the levels of proapoptotic proteins BIM and PUMA, which are members of BH3 family of proteins that regulate cellular apoptosis^37,38^ (Fig. 6b, c). Further, we observed elevated levels of the transcription factor FOXO1, that is known to target BIM and PUMA during apoptosis^39–45^. Importantly, increased levels phosphorylated Smad3 protein were also observed in palmitate-treated MIN6 cells and mouse islets (Fig. 6b, c). We next examined potential interactions between the TGF-β/Smad3 and FOXO1 signaling pathways during palmitate-induced β-cell apoptosis. Coimmunoprecipitation analyses revealed interaction between Smad3 and FOXO1 proteins in MIN6 cells stimulated by TGF-β and palmitate (Fig. 6d, e). This interaction was undetectable in the presence of a small molecule TβR1 kinase inhibitor, SB431542, suggesting a role for TβR1 activity in the interaction. Together, these results are evidence of a TGF-β–Smad3–FOXO1-mediated β-cell apoptosis program in response to metabolic stress.

### Protection from β-cell apoptosis by inhibition of TGF-β/Smad3 and FOXO1 signaling

**Fig. 7:**
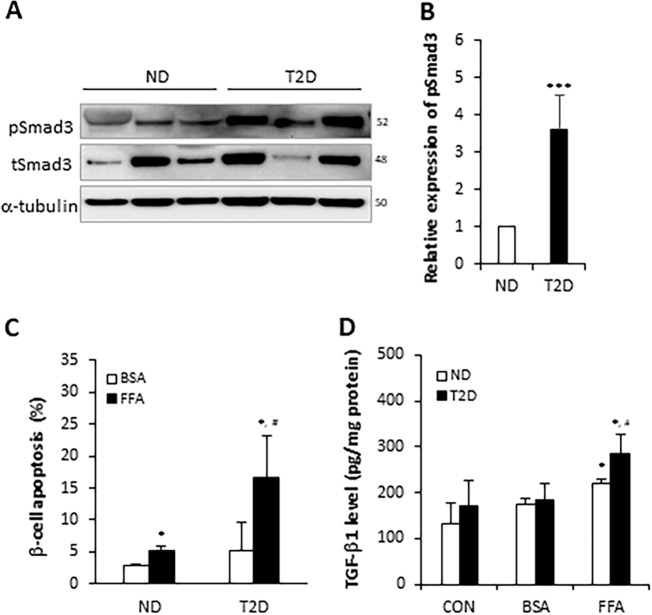
Activation of TGF-β signaling during FFA-induced β-cell apoptosis in human islets. **a** Islets from three individual nondiabetic (ND) and T2D human islet cadaver donors were monitored for activation of TGF-β signaling by evaluating pSmad3 expression in western blotting assays. **b** pSmad3 expression was normalized to tubulin expression and presented as fold change. **c** Islets (*n* = 3 donors) were dispersed by trypsin and cultured with 0.5 mM PA or 1% BSA as vehicle control for 24 h. Subsequently, the islet cells were stained with TUNEL and insulin for detection of β-cell apoptosis. **d** 15 islets from each of the three ND and T2D cadaver donors were treated with free fatty acid (FFA) using 0.5 mM of PA or vehicle (1% BSA) in 0.3 ml of 2% charcoal-treated FBS containing CMRL 1066 media for 24 h and media were collected for TGF-β1 measurement. The concentration of TGF-β1 in cultured media was normalized to total protein amount in each 15 islets (*n* = 3 replicates per donor). ***p < 0.001 vs. ND (**b**); *p < 0.05 vs. BSA; ^#^*p* < 0.05 vs. ND (**c**, **d**).

Butler et al. observed increased β-cell apoptosis in cadaver pancreas from human T2D subjects, thus suggesting a role for apoptosis as an important mechanism responsible for the reduced β-cell mass in diabetes^15^. Thus, we examined the status of TGF-β signaling in islets from ND and T2D human cadaver donors (Supplementary Table 1). Western blot analyses revealed significant elevation of phospho-Smad3 in T2D islets (Fig. 7a, b). Next, ND and T2D human cadaver donor islets were cultured in the presence or absence of palmitate (free fatty acid (FFA)) and the levels of β-cell apoptosis was measured. Quantifying numbers of TUNEL^+^insulin^+^ cells demonstrated significantly increased levels of FFA-stimulated β-cell apoptosis in T2D islets compared with that seen in ND human cadaver donor islets (Fig. 7c). Islets exposed to FFA secreted elevated levels of TGF-β1 ligand (Fig. 7d). Moreover, levels of secreted TGF-β1 were higher in FFA-stimulated human T2D islets (Fig. 7d), that further suggest a stimulatory effect of TGF-β signaling during β-cell apoptosis. Taken together with the elevated phospho-Smad3 levels in T2D islets (Fig. 7a, b), these findings are consistent with an autocrine role for TGF-β1 in β-cell apoptosis.

**Fig. 8:**
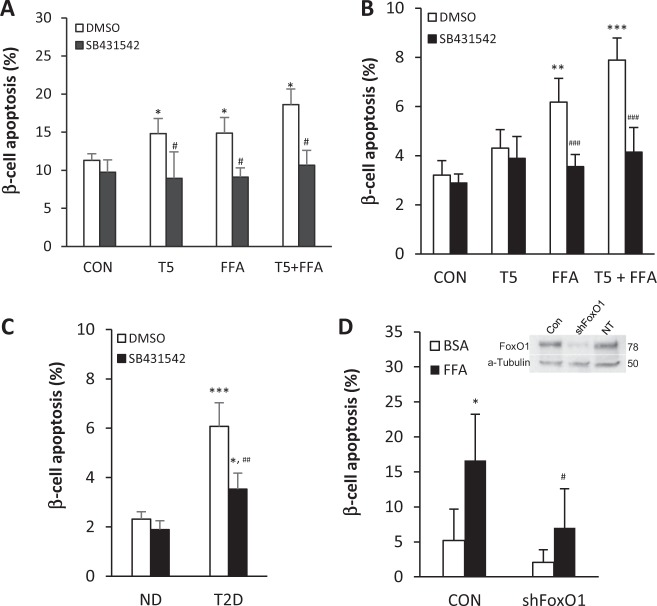
Protection from β-cell apoptosis by inhibition of FoxO1 or TGF-β/Smad3 signaling. Cultured islets from (**a**) normal wild-type mice (*n* = 3) or (**b**) nondiabetic (ND) human cadaver donors (*n* = 3) were treated with either 5 ng/ml TGF-β1 (T5) or 0.5 mM PA (FFA) or combination of TGF-β1 and PA (T5 + FFA) with or without 10 µM SB431542 for 24 h. DMSO was used as vehicle control for SB431542. **c** Islets from ND and T2D donors (*n* = 3 each) were treated with 10 µM SB431542 for 24 h. **d** Islets from T2D donors (*n* = 2) were infected with nontarget control (CON) or shFoxO1 lentivirus for 72 h and then treated with 0.5 mM PA (FFA) or 1% BSA for additional 24 h. Inset shows decreased FOXO1 expression in islets after 72 h of shFoxO1 lentivirus infection. After all the above-mentioned treatments the islets were fixed and stained with TUNEL and insulin for β-cell apoptosis assessment. The number of TUNEL+ insulin+ cells were divided by total number of insulin+ cells and the β-cell apoptosis rate was calculated and presented as % of β-cell apoptosis. *p < 0.05, **p < 0.01, ***p < 0.001 vs. CON or ND; ^#^*p* < 0.05, ^##^*p* < 0.01, ^###^*p* < 0.001 vs. DMSO (**a–c**); *p < 0.05 vs. BSA, ^#^*p* < 0.05 vs. CON (**d**).

Finally, we inquired if inhibition of TGF-β signaling would reduce the susceptibility to β-cell apoptosis. Mouse and human cadaver islets were incubated with TGF-β or FFA alone or in combination, with or without the addition of SB431542, followed by monitoring for β-cell apoptosis. Incubation with TGF-β ligand or FFA alone resulted in increased β-cell apoptosis in normal mouse islets (Fig. 8a) and ND human cadaver donor islets (Fig. 8b). Combination of TGF-β and FFA led to further elevation of β-cell apoptosis (Fig. 8a, b). Importantly, treatment with SB431542 resulted in suppression of β-cell apoptosis, thus confirming that TβR1 activation is essential for β-cell apoptosis (Fig. 8a, b). Treatment with SB431542 lowered the levels of apoptosis to that of control levels demonstrating a strong protective effect due to inhibition of TGF-β signaling. Importantly, T2D human islets with high basal levels of β-cell apoptosis were significantly protected by addition of SB431542 (Fig. 8c). We also examined levels of FFA-induced β-cell apoptosis in islets isolated from *Smad3*-deficient mice (*Smad3* KO)^29^. Compared with a significant increase in β-cell apoptosis in control *Smad3* WT islets, the *Smad3* KO islets were protected from FFA-induced β-cell apoptosis (Supplementary Fig. 4b). Finally, culture of human T2D islets in the presence of a small hairpin RNA targeting FOXO1 (shFoxo1) significantly reduced the levels of FFA-induced β-cell apoptosis (Fig. 8d), thus indicating an important role for FOXO1 in β-cell apoptosis. Taken together, these data support the notion that increased TGF-β/Smad3 signaling stimulates, whereas inhibition of TGF-β/Smad3 signaling protects from β-cell apoptosis.

## Discussion

The β-cell is a prime target in the complex etiology of T2D diabetes pathogenesis. β-cell dysfunction and severe deficits to β-cell mass underlie the β-cell failure commonly seen in T2D. In agreement, genome-wide association studies of T2D show enrichment of genes associated with β-cell biology^46–48^. Plethora of studies have described the mechanisms of β-cell dysfunction in T2D^49^. Similarly, research into β-cell mass regulation has yielded information relevant to understanding β-cell proliferation and regeneration^50,51^. Thus, β-cell replication and neogenesis from facultative endocrine progenitors appear to be the main mechanisms that increase β-cell mass. However, rates of β-cell proliferation are minimal in the adult pancreas and the capacity to regenerate new β-cells is known to diminish with age and under sustained metabolic stress^52,53^. Although, β-cell apoptosis has been observed in T2D human cadaver pancreas^15^, there is insufficient mechanistic insight into the process.

Here, we report that TGF-β/Smad3 signaling promotes β-cell apoptosis. Activation of TGF-β/Smad3 signaling correlates with increased apoptosis in islets from HFD-fed mice and cadaveric human T2D islets. Mice with β-cell-specific constitutively-active TGF-β/Smad3 signaling display increased susceptibility to β-cell apoptosis, which is associated with glucose intolerance, β-cell dysfunction, reduced β-cell mass, and diabetes. In contrast, mice with inhibited Smad3 signals in β-cells are protected from β-cell apoptosis and exhibit improved glucose tolerance with enhanced β-cell mass and function. A limitation to the studies is the transgene design which does not include a tag in order to identify or locate the expressed transgenic proteins. This precludes determining (i) the relative levels of transgene expression in β-cells, (ii) the percentage of β-cells expressing the transgenes upon Dox administration during embryonic development or adult stages, and (iii) if the differences in phenotypes observed in adult vs. embryonic Dox-based transgene activation are due to variations in transgene expression.

FFAs induce significant increases in β-cell apoptosis which is augmented by activation of TGF-β signaling. In contrast, genetic deletion of Smad3 or pharmacological inhibition of TGF-β signaling via a small molecule TβR1 kinase inhibitor, SB431542, protects from FFA-stimulated β-cell apoptosis. We demonstrate that FFA-stimulated T2D islets secrete significant levels of TGF-β ligand and exhibit elevated phospho-Smad3 expression, which is consistent with an autocrine role for TGF-β in promoting β-cell apoptosis. Further, we observed that FFA treatment increased the levels of proapoptotic Bcl2 family proteins, BIM and PUMA, which are targets of the FOXO1 transcription factor. We observed association of Smad3 with FOXO1 upon treatment with TGF-β and palmitic acid, with that interaction abolished in the presence of the TβR1 kinase inhibitor SB431542. Together, these results suggested that FFAs stimulate the TGF-β/Smad3 network to promote β-cell apoptosis via FOXO1 and Bcl-2-family proteins.

The increased levels of pSmad3 observed in the T2D subjects is probably due to chronic exposure of islets to the circulating lipid levels and the autocrine TGF-β signals from T2D islets. In Fig. 7c, we observed marginal but variable increase in apoptosis in the BSA-treated T2D islets possibly reflecting differences intrinsic to the cadaver donors. We surmise that the T2D islets (like the CA-Smad3 mouse islets) are susceptible to apoptosis and have the apoptosis machinery activated as a result of constitutively-active TGF-β signaling, evidenced by high levels of pSMAD3 in the T2D human islets and the CA-Smad3 mouse islets. The same scenario is also likely in the HFD-fed mouse islets. The overactive TGF-β/pSMAD3 signals in the HFD islets prepare the cell death machinery to undergo apoptosis. The addition of HFD, FFA, or presence of a diabetic environment in the case of human subjects initiates the apoptosis process. Autocrine islet TGF-β signaling further propagates β-cell apoptosis. Taken together, these observations are consistent with the notion that TGF-β/SMAD3 signals are essential to initiate the apoptosis program. Propagation of the apoptosis program further requires autocrine TGF-β/pSMAD3 signaling in conjunction with accessory signals, like FFA stimulation, to amplify the apoptosis signal.

The roles of extracellular growth factors in β-cell apoptosis is largely obscure. Garcia-Ocana et al. reported a complex role for hepatocyte growth factor (HGF) signaling in β-cell apoptosis^54,55^. A recent study reported the role of growth differentiation factor 11 (GDF11) in β-cell apoptosis via actions on TGF-β/Smad2 signaling^56^. Activin receptor-like 7 (ALK7) has also been linked to apoptosis via Smad2 and Caspase-3^30,57^. The source of HGF, GDF11, or ALK7 during the β-cell apoptosis program is unknown, and it is unclear if these ligands operate via autocrine or paracrine mechanisms. We find that TGF-β1 is released from islets and that its levels are augmented when islets are exposed to FFAs, thus supporting an autocrine role for TGF-β1 ligand during β-cell apoptosis. Elevated TGF-β signaling—as evidenced by increased phospho-Smad3 levels—is observed in islets isolated from HFD-fed mice and T2D human cadaver islets. While we infer that FFA signals promote autocrine TGF-β1 secretion from β-cells within the islets thus activating Smad3 signaling, we cannot exclude the possibility that other cells within the islets or within the pancreatic milieu serve as a paracrine source for TGF-β’s actions. Indeed, circulating levels of TGF-β1 are increased in human T2D patients^58,59^ and in HFD-fed mice^60^, thus allowing for the possibility of paracrine modes of activation of Smad3 signaling.

The findings that human T2D cadaveric pancreas exhibit elevated β-cell apoptosis, suggest that developing methods to slow down or halt the apoptosis program may be therapeutically beneficial. Studies have shown that glucagon-like peptide 1 and its small molecule analog, liraglutide, prevents FFA-induced β-cell apoptosis^61,62^. Our findings that a small molecule inhibitor of the TβR1 kinase, SB431542, protects T2D human islets from β-cell apoptosis supports the notion that controlled blockade of TGF-β/Smad3 signals may indeed be beneficial. The observation that DN-Smad3 mice exhibit increased β-cell mass and function, have improved GSIS, and enhanced glucose tolerance is consistent with this idea. The observation that SB431542 can significantly block β-cell apoptosis in normal mouse and ND human islets as well as in human T2D islets is of translational relevance for diabetes. TGF-β’s role in regulating apoptosis in normal and diseased cells is well known^63^. We have previously shown that TGF-β antagonism can protect mice from obesity and diabetes^60,64^. The findings here support a role for controlled TGF-β inhibition in combating β-cell apoptosis and restoring the β-cell mass in diabetes.

## Materials and methods

### RD and HFD mice

Age-matched C57BL/6J male mice were purchased from Jackson Laboratory (Bar Harbor, ME, USA). Mice were either fed RD or 60% kcal HFD (Research Diets, New Brunswick, NJ, USA) and body weight measurements, glucose level determinations, glucose tolerance assays, and ex vivo GSIS assays were performed. Pancreatic sections and isolated islets were tested for levels of β-cell apoptosis and TGF-β/Smad3 signal levels as described below. TGF-β1 level was measured in fed state plasma using the TGF-β1 ELISA kit (R&D, Minneapolis, MN, USA).

### Transgenic mice and activation of transgenes

We used two previously established lines, RIP7-rtTA mice (The Jackson Laboratory, Bar Harbor, ME, USA), and TRE-Smad3 mice^65^ expressing WT Smad3, constitutively-active (CA) Smad3 (with the last two serines substituted by aspartic residues to mimic phosphorylation) and DN Smad3 (lacking the last four amino acid residues) via the TRE. Pancreas-specific expression of the Smad3 transgenes was achieved by crossing each of the TRE-Smad3 mice lines with RIP7-rtTA mice, that express reverse tetracycline-controlled transactivator (rtTA) specifically in β-cells under the control of the rat insulin II promoter (RIP7). The resulting double transgenic mice were designated as R7:WTS3, R7:CAS3, and R7:DNS3, respectively. The expression of Smad3 transgenes was induced by feeding Dox diet (Dox, 200 mg/kg body weight, Bioserv, Frenchtown, NJ, USA) as follows until further analyses were performed: (1) embryonic activation: the breeding pair and their offspring were fed the Dox diet, and (2) adult activation: adult mice of different ages were fed the Dox diet for designated time period. For adult HFD challenge study, 2-month-old adult mice were fed 60% HFD supplemented with 200 mg/kg Dox until the time when analyses were performed. All mice were maintained and handled according to protocols approved by the NIH Animal Care and Use Committee.

### Glucose homeostasis and GSIS

Long-term fasted (16 h overnight fast) or daytime short-term fasted (8am to 2 pm fast) or morning-fed blood glucose and plasma insulin levels were measured from male mice at indicated time point. GTT was performed in 2 months old male mice following embryonic transgene activation. Mice were intraperitoneally injected with glucose (2 g/kg body weight). Blood glucose levels were measured at 0, 15, 30, 60, and 120 min after glucose injection with AlphaTRAK2 glucometer (Abbott, Abbott Park, IL, USA) and plasma insulin levels were measured by the ELISA kit (ALPCO, Salem, NH, USA) at 0 and 30 min after glucose injection using blood drawn from tail vein. The AUC was calculated. Ex vivo GSIS assays were performed in isolated islets from RD- and HFD-fed male mice. After isolation, ten similar sized islets from each group were handpicked under a stereoscope and used for GSIS. The islets were cultured in Krebs–Ringer bicarbonate buffer (KRBB) without glucose for 1 h at 37 °C and then stimulated with KRBB containing either 2.8 mM or 16.7 mM glucose for another 1 h. After 1 h glucose stimulation, the buffer was collected to check levels of GSIS and the islets were harvested for protein extraction. GSIS values are presented after normalizing to the total cellular protein content in each ten islets.

### Islet isolation, dissociation, and detection of apoptosis

Mouse islets were isolated from pancreas tissue via digestion with collagenase type 1A (1 mg/ml, Sigma, St. Louis, MO, USA) and purified using Histopaque (Sigma) gradient, followed by handpicking under a stereomicroscope and culture in RPMI with 10% FBS. Identical number of similarly sized islets were used for GSIS assays. For detecting β-cell apoptosis, islets were dissociated by incubation with 0.05% trypsin for 5 min at 37 °C. Dispersed islet cells were cultured in chamber slides (Millipore Sigma, Burlington, MA, USA) overnight. The next day dispersed islet cells were treated as indicated for each experiment. After treatment, the islet cells were fixed with 4% paraformaldehyde and stained with antibodies.

### Immunohistochemistry and immunofluorescence

Pancreas were harvested and fixed in 10% buffered formalin. Paraffin sections were immunostained with the following primary antibodies overnight at 4 °C: guinea pig anti-insulin (Dako, Carpinteria, CA, USA), rabbit anti-cleaved caspase-3 (Cell signaling, #9664), mouse anti-BrdU (BD Bioscience), and rabbit anti-Smad3 (Abcam, Cambridge, MA, USA). Following primary antibody incubation, either ABC system (Vector Lab, Burlingame, CA, USA) was used for histochemistry development, or secondary antibodies coupled to Alexa-488, Alexa-568, and Alexa-647 (Molecular Probes, Eugene, OR, USA) were used in immunofluorescence staining. For apoptosis, the DeadEnd™ Fluorometric TUNEL (Promega, Madison, WI, USA) assay kit was used according to the manufacturerʼs instructions. For BrdU staining, the mice were injected intraperitoneally with BrdU (30 mg/kg body weight, Sigma) 2 h before euthanasia.

### Morphometric analysis

For each pancreas, at least three inconsecutive sections with 150 µm separation were randomly chosen for staining per mouse. For determination of the β-cell area, entire pancreatic sections were immunostained for insulin using peroxidase indirect labeling technique. The area of insulin-positive cells, as well as that of total pancreatic sections, were evaluated in each stained section image captured (Olympus VS120 Slide Scanner microscope, Shinjuku, Tokyo, Japan) and analyzed using image analysis ImageJ software (NIH, Bethesda, MD, USA). The β-cell area (%) occupied in the total pancreatic tissue area was determined. Results represent the average from four animals per group. For apoptosis, the β-cell apoptosis rate was evaluated as % of total TUNEL+ β-cells in total β-cells. At least 3000 β-cells were counted per animal and at least three animals per group were used for proliferation and apoptosis study.

### Human islet studies

Islets from human cadaver donor pancreas were obtained via the NIDDK-sponsored Integrated Islet Distribution Program, CA. Details regarding the human islet donors used in this study are provided (Supplementary Table 1). After overnight recovery at 37 °C, islets were handpicked and used for western blot analyses and the TGF-β1 secretion assay. Similar sized 15 islets per well were treated with 0.5 mM palmitate (PA, Sigma) in 2% charcoal-treated FBS containing CMRL 1066 media (Gibco, ThermoFisher Scientific, Waltham, MA, USA) for 24 h and the TGF-β1 level measured in the media was normalized to protein extracted from 15 islets. For the apoptosis detection, handpicked islets were dissociated with trypsin at 37 °C and dispersed islets were used. Lentiviral particles containing nontargeting short hairpin RNA (CON) or shRNA against to FoxO1 (shFoxO1) were purchased from Dharmacon (Lafayette, CO, USA). The sequence of FoxO1 shRNA was 5′ -ACATATGGCCAATCCAGCA-3′. Dissociated T2D human islet cells were infected with control or shFoxO1 lentiviral particles with polybrene (8 µg/ml) for 3 days and subsequently the islet cells were treated with for FFA palmitate or SB431542 (Sigma) for additional 24 h.

### FFA preparation and treatment

For FFA treatment, we used palmitate as FFA source for MIN6 cells and islets. Bovine serum albumin (BSA)-conjugated palmitate solutions were prepared as described previously^66^. For most experiments, 0.5 mM palmitate complexed with 1% fatty acid free BSA was used for FFA treatment and 1% BSA was used as vehicle control.

### MIN6 cells, transfection, and immunoprecipitation

MIN6 cells were cultured at 37 °C in high glucose (5.5 mM) DMEM supplemented with 10% fetal bovine serum and 1% penicillin/streptomycin. For exogenous coimmunoprecipitation experiments, MIN6 cells were transfected with Flag-tagged WT Smad3 expression plasmid^67^ using lipofectamine 2000 (Invitrogen, Carlsbad, CA) for 48 h and the cells were treated with 5 ng/ml of TGF-β1 and 0.5 mM PA with or without 10 µM of SB431542 (SB) for additional 24 h. After treatment, the MIN6 cells were lysed in immunoprecipitation buffer using a Pierce coimmunoprecipitation assay kit (Pierce Biotechnology Ltd., Rockford, IL, USA) following manufacturerʼs instructions. Immunocomplexes were determined using specific antibodies as indicated.

### Immunoblotting

Protein samples were prepared from isolated islets and MIN6 cells in RIPA lysis buffer with protease and phosphatase inhibitors (Sigma). Proteins were resolved on Tris/glycine gel and transferred to PVDF membrane (Bio-Rad, Hercules, CA, USA). The following primary antibodies were incubated overnight at 4 °C: rabbit anti-Smad3 (1:1000, Abcam, ab28379), mouse anti-Smad3 (1:1000, Abcam ab75512), p-Smad3 (1:1000, Abcam, ab52903), FOXO1 (1:1000, Cell signaling, Danvers, MA, #2880), p-FOXO1 (1:1000, Cell signaling, #9461), PUMA (1:1000, Cell signaling, #4976), BIM (1:1000, Cell signaling, #2933), α-tubulin (Sigma, T5168), β-actin (Cell Signaling, #3700), and Flag (Sigma, F1804). Secondary antibodies horseradish peroxidase-conjugated anti-rabbit and anti-mouse (Santa Cruz, Dallas, TX, USA) were used. Proteins were detected using ECL plus substrate (Thermo Fisher) and images were acquired on ChemiDoc Touch Imaging System (Bio-Rad). Densitometry analysis was performed using Image Lab software (Bio-Rad).

### Statistical analysis

All data are presented as mean ± SD. Statistical significance was determined using two-tailed and nonpaired Student’s *t* test and the level of significance was set at *p* < 0.05, 0.01, or 0.001.

## Supplementary information

Supplementary Table 1

Supplementary Figure 1

Supplementary Figure 2

Supplementary Figure 3

Supplementary Figure 4
