# Supplementary material for: Protection from β-cell apoptosis by inhibition of TGF-β/Smad3 signaling
Source: Cell Death Dis. 2020 Mar 13;11(3):184. doi: 10.1038/s41419-020-2365-8 (PMC7070087; doi:10.1038/s41419-020-2365-8)
Supplement: Supplementary file 1 — Supplementary Table 1 [file 41419_2020_2365_MOESM1_ESM.docx]

| Parameter | **Non-diabetic subjects** | | | | | **Type 2 diabetic subjects** | | | | |
| --- | --- | --- | --- | --- | --- | --- | --- | --- | --- | --- |
| Donor ID | 1868 | 1923 | 1997 | 3003 | SAMN  09479728 | 1866 | 1888 | 1981 | 1957 | SAMN  09239001 |
| Age (yr) | 38 | 57 | 45 | 29 | 41 | 46 | 59 | 45 | 37 | 50 |
| Gender | M | F | M | M | F | F | M | M | F | M |
| BMI | 25.5 | 27.5 | 26.6 | 23 | 25 | 35.9 | 27.7 | 27.2 | 38.1 | 36.2 |
| Ethnicity | Hispanic/  Latino | White | Hispanic/  Latino | Hispanic/  Latino | White | White | Hispanic/  Latino | White | White | Hispanic/  Latino |
| Cause of Death | Cerebrovascular/stroke | Anoxia | Cerebrovascular/stroke | Head trauma | Cerebrovascular/stroke | Cerebrovascular/stroke | Cerebrovascular/stroke | Cerebrovascular/stroke | Cerebrovascular/stroke | Cerebrovascular/stroke |

**Supplementary Table 1. Human pancreatic islet donor information**

Islets were provided by the Integrated Islet Distribution Program, City of Hope.
