# Supplementary figures and images for: Protection from β-cell apoptosis by inhibition of TGF-β/Smad3 signaling
Source: Cell Death Dis. 2020 Mar 13;11(3):184. doi: 10.1038/s41419-020-2365-8 (PMC7070087; doi:10.1038/s41419-020-2365-8)

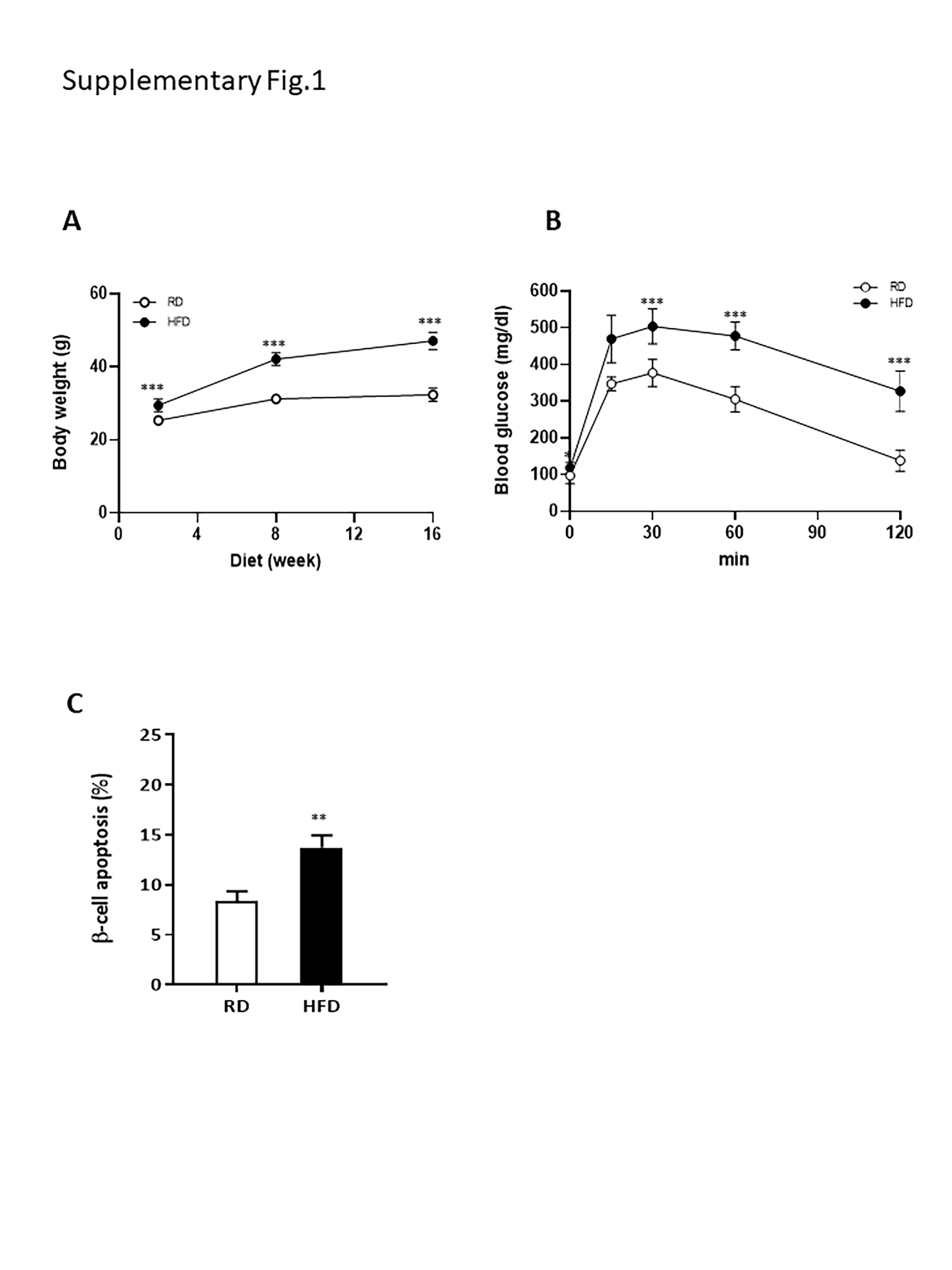

Supplement: Supplementary file 2 — Supplementary Figure 1 [file 41419_2020_2365_MOESM2_ESM.tif]

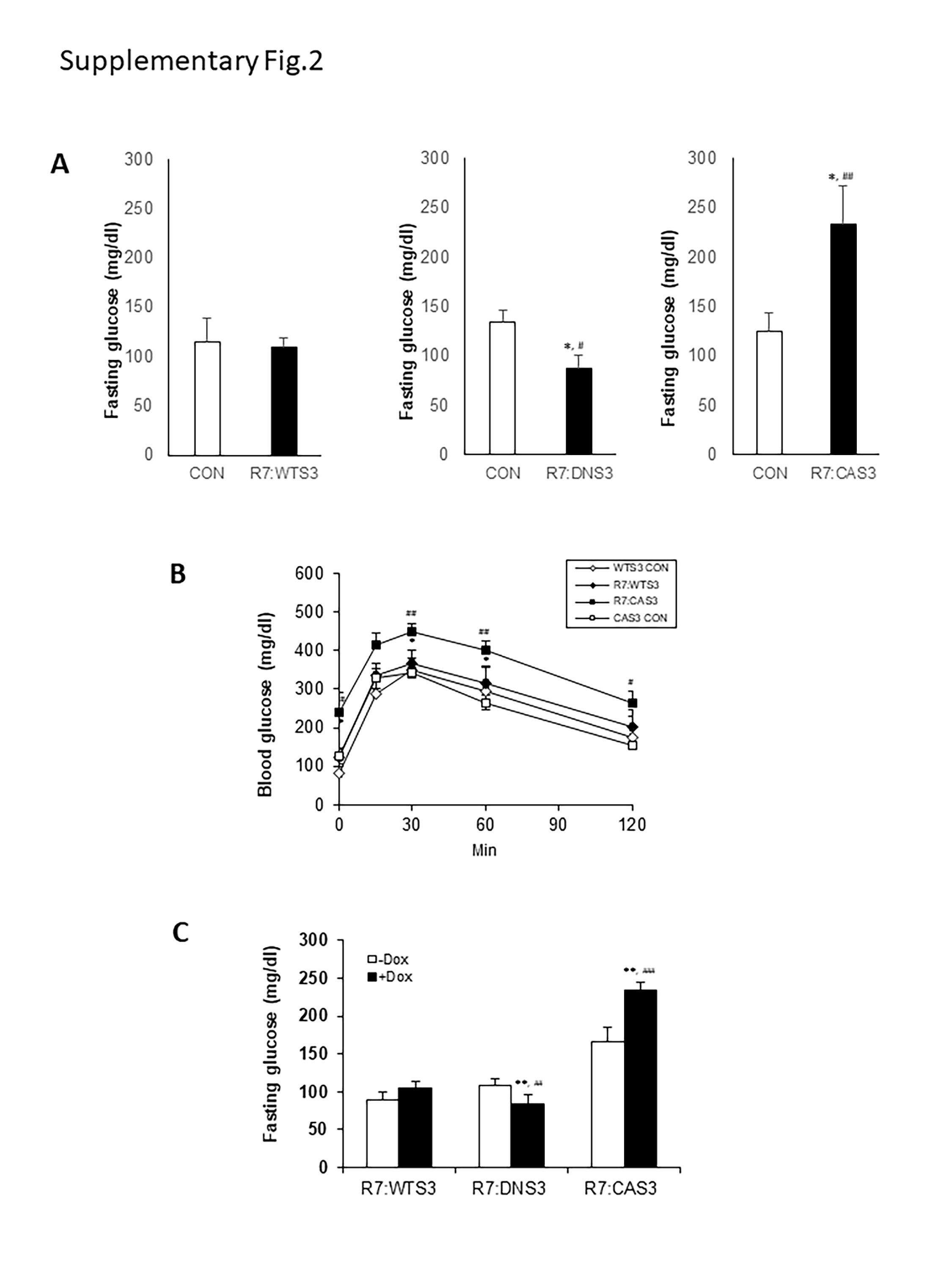

Supplement: Supplementary file 3 — Supplementary Figure 2 [file 41419_2020_2365_MOESM3_ESM.tif]

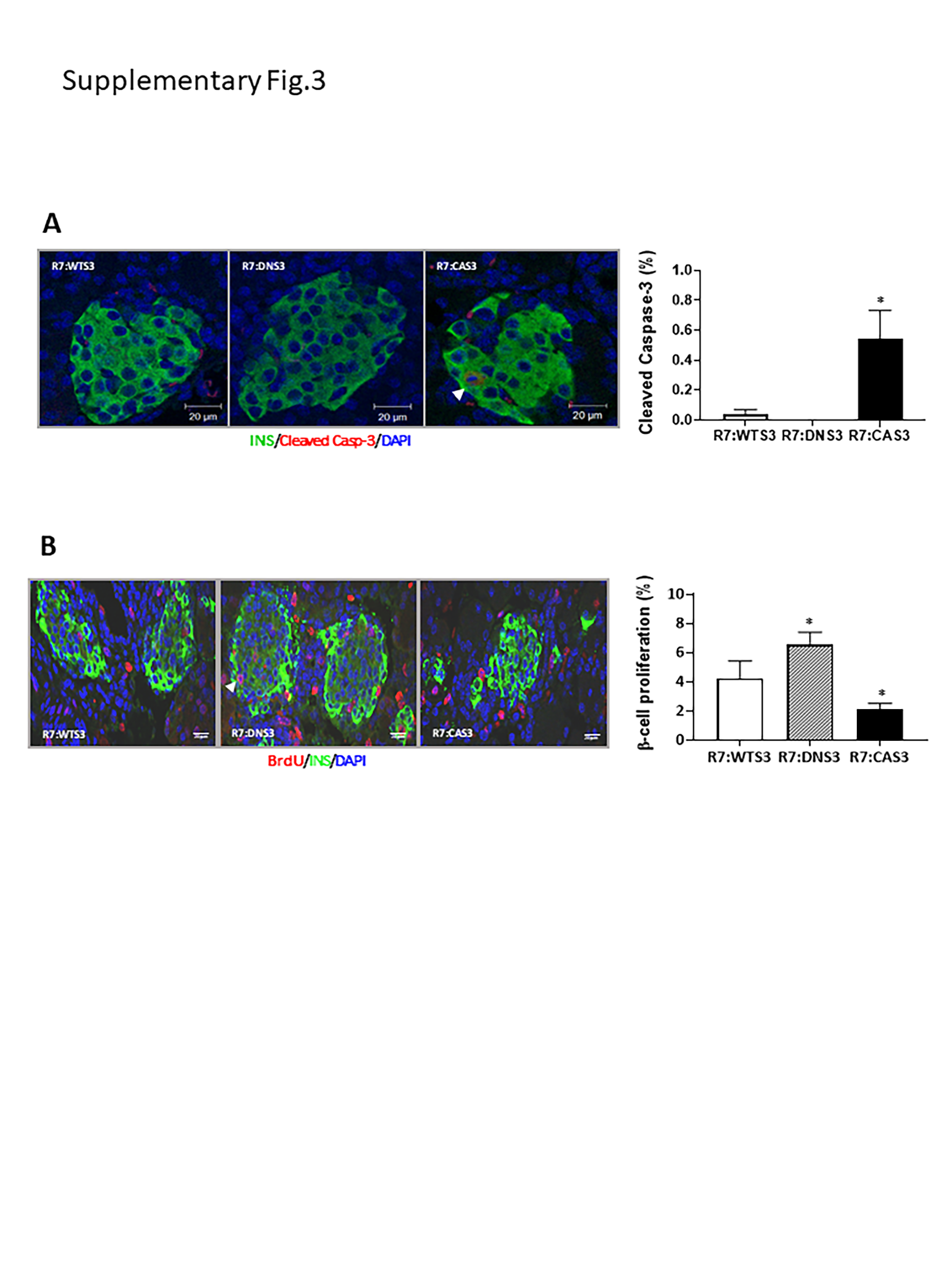

Supplement: Supplementary file 4 — Supplementary Figure 3 [file 41419_2020_2365_MOESM4_ESM.tif]

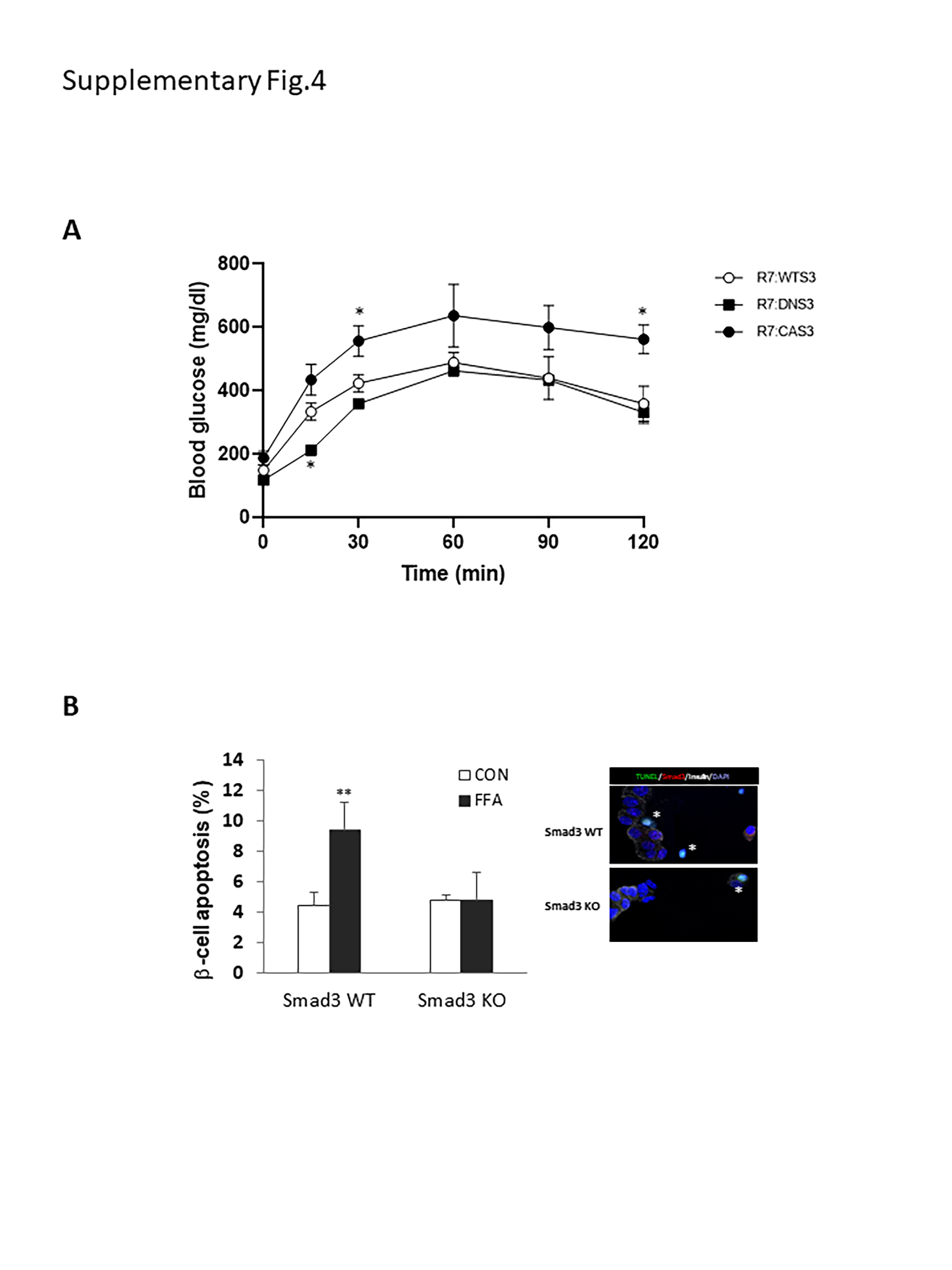

Supplement: Supplementary file 5 — Supplementary Figure 4 [file 41419_2020_2365_MOESM5_ESM.tif]
